# Enhancing the valorization of pulping black liquors in production effective aerogel–carbon nanostructure as adsorbents toward cationic and ionic dyes

**DOI:** 10.1038/s41598-024-65136-8

**Published:** 2024-07-02

**Authors:** Vivian F. Lotfy, Altaf H. Basta

**Affiliations:** Cellulose and Paper Department, National Research Centre, Dokki, Giza 12622 Egypt

**Keywords:** Rice straw-pulping black liquors, Safety disposing, New aerogels, Effective dyes adsorbents, Adsorption models and kinetic, Environmental sciences, Chemistry, Materials science, Nanoscience and technology

## Abstract

This work deals with promoting the efficiency of removing the cationic and ionic dyes by new aerogel–carbon nanostructures. For cleaner production the rice straw-pulping black liquors, which regards serious environmental risk during routine disposing, is used in preparing the aerogel precursors. These aerogels (AGBs) depend on using pulping black liquor in hybrid with resorcinol and the less carcinogenic formaldehyde butyraldehyde. Black liquors from five pulping processes are used, Elemental, thermogravimetric (TGA and DTG), and FTIR-ATR analyses are used to characterize the carbon precursors. While their adsorption behavior toward cationic and anionic dyes are accessed via iodine-value, adsorption capacity and kinetic models, textural characterization, and SEM. The TGA measurements reveal that AGBs from BLs of neutral sulfite and soda-borohydride pulping reagents have higher activation and degradation energies than other aerogels. In terms of cationic and anionic dyes adsorption as well as textural characterization, the AGB-CNSs surpass that made from BLs. The discarded KOH/NH_4_OH black liquor is used to synthesize the best aerogel precursor for producing cationic methylene blue dye (MB) adsorbent, where it provides an adsorption capacity 242.1 mg/g. The maximum anionic brilliant blue dye (BB) adsorption capacity, 162.6 mg/g, is noticed by Kraft BL-aerogel-CNSs. These finding data overcome the literature carbon adsorbents based on lignin precursors. All examined CNSs toward MB dye follow the Langmuir adsorption equilibrium; while primarily the Freundlich model for BB dye. The pseudo-second-order kinetic model well fits the adsorption kinetics of investigated AGB-CNSs. The textural characterization and SEM revealed a mixture of mesoporous and micro porous features in the CNSs.

## Introduction

Dyes are organic-colored compounds extracted from plants, animals, and leaves. Natural dyes were discovered first, but synthetic dyes were discovered by English chemist W.H. Perkin in 1985^1^. Chemical bonding is used to apply colors to substrates like textiles, paper, and leather^2,3^. These dyes are widely used in industry but can have negative environmental and human health effects. Most are poisonous, cancer-causing, and mutagenic to aquatic creatures, leading to water pollution^4,5^. Various strategies and methodologies are being investigated to remove dyes using physical or chemical methods^6,7^.

Methylene blue, a redox dye from the phenothiazinium family, is used in operating rooms and intensive care units for treating various ailments and staining. It has anti-inflammatory, anti-malarial, antidepressant, and cardioprotective properties^8–10^. Methylene blue, a cationic dye, is a well-known water contaminant due to its industrial applications^6,11^. Coomassie Brilliant Blue R-250 (CBB) dye, an essential anionic dye in the textile industry, is a harmful and resistant organic pollutant. It is commonly used to stain proteins in clinical and biochemical laboratories^12^. It is triphenylmethane dyes, which are frequently used to stain and evaluate proteins in clinical and biochemical laboratories. This dye is resistant to acidic environments, heat, and light. The non-biodegradable nature of CBB it is dangerous to the environment and human health and irritates the eyes, respiratory system, and gastrointestinal tract^13^.

Removing and decreasing the concentration of MB and CBB dyes in wastewater in order to reduce environmental contamination is necessary. To eliminate toxic chemicals and protect the environment, several techniques have been used, such as adsorption^14–16^, membrane-based separation^17^, solvent extraction^18^, flocculation^19^, and ultra-filtration^20^ are a few of the methods used to treat dye-contaminated wastewater. The adsorption technique is one of the most appealing and adaptable routes because of its efficacy, simplicity, and low cost. Many different adsorbents have been used in wastewater treatment, including mesoporous silica, polymer resin and carbon-based nanomaterials^21^. Due to their high porosity, carbon-based nanomaterials have a great deal of potential for use in environmental applications as effective adsorbents. It has also received a lot of attention in the fields of optical materials, energy storage, and biomedical applications^22–25^.

Black liquor is produced as a byproduct of the pulp and paper industry. One of the problems facing the agro-waste paper pulping industry is the discharge of black liquor (BL). Recent developments have been applied in the conversion of BL into fuels and chemicals by biological and chemical processes. Durmaz et al., Fang et al., and Sathya et al.^26–28^, reported that BL or BL-lignin is used to produce wood preservatives, resin, and paint, as well as dispersing and stabilizing agents for dyes, control release systems for agriculture, and carbon precursors.

In this current study the effective performance of black liquor-based new aerogels as precursors for carbon manufactures, is assessed via comparing their affinity to adsorb the cationic and anionic dyes versus those produced from black liquors. The role of the rice straw pulping agents (sodium hydroxide, sodium hydroxide-borohydride, potassium hydroxide/ammonium hydroxide, neutral sulfite, and Kraft) on the dyes' adsorption process is examined. Carbon precursors (BLs or BL-aerogels) are subjected to elemental analysis, thermal analysis and FTIR analysis. Actionably, the performance of different carbon nanostructures (cationic and anionic adsorption behavior, FTIR, textural characteristics, and SEM) will be compared with that of earlier carbon-based nanomaterials.

## Materials and methods

### Materials

Black liquors (BLs), which stand for carbon precursors, were obtained through pulping rice straw (RS), an agricultural waste product utilized in Egypt. El-Nasr Pharmaceutical Chemical Co. (ADWIC) supplied the pulping reagents utilized in the pulping process, which included sodium hydroxide, potassium hydroxide, sodium sulfite, and sodium carbonate (C) as well as resorcinol B.P 93 (R). In addition to ammonium hydroxide (25%) from BDH Limited in Germany, Riedel-de Haan AG in Seelze Hannover provided sodium sulfide and sodium borohydride (BH). The butyraldehyde solution (BA), 98% were supplied from Alfa AesarGmbh & Co KG. Phosphoric acid, 85%, was supplied for activation by El-Nasr pharmaceutical chemistry Co. (ADWIC). Methylene blue dye (MB), Coomassie brilliant blue R-250 (CBB) and iodine were acquired from Alfa Chemicals Co., SD fine-chem Ltd (India), and El Nasr Pharmaceutical Chemical as the adsorbate for measuring the adsorption performance of carbon-based products.

**Table:** 

Dye	Concentration, ppm	Maximum wavelength, nm	Molecular weight, g/mol	Color index (CI) number
Methylene blue (MB)	1000	664	319.85	52,015
Coomassie brilliant blue R-250 (CBB)	1000	553	825.97	42,660

### Preparation of Black liquors

Rice straw (RS) was pulped using a variety of reagents, including sodium hydroxide without or with addition (sodium borohydride [BH]), potassium hydroxide/ammonium hydroxide, neutral sulfite, and Kraft pulping conditions. The following operating parameters were used for the pulping processes: 6:1 liquor ratio for 1 h at 120 °C in autoclaves. For NaOH pulping without and with 3% BH], 18.6% Na_2_O is comparable. The black liquor produced by pulping soda was marked with the following codes: BL1 for NaOH pulping and BL2 for NaOH-3%BH pulping. The BLs produced by potassium hydroxide/ammonium hydroxide mixture in ratio 1:5, neutral sulfite pulping (mixture of sodium sulfite and sodium carbonate with mass ratio 4:1 and the Kraft method by using 10% active alkali and 25% sulfidity, were designated BL3, BL4, and BL5, respectively.

Based on the Dumas combustion method, the Profilenc Technologies ECS 8020 CHNS-O elemental analyzer (Italy) was used to identify the main elements (C, N, H, S, and O) of the produced BLs. It represents a development of chromatographic separation and sample combustion-based elemental analysis methods.

### Synthesis of BL-resorcinol butyraldehyde carbon aerogels (AGBs)

The preparation of organic gel, in absence of black liquors, was depended on replacing of carcinogenic formaldehyde with long chain aldehyde called butyraldehyde to form resorcinol (R)/butyraldehyde (BA) aerogel (AGB). The AGB was synthesized under the following circumstances: the R/BA molar ratio equal to stoichiometric (0.5), resorcinol/carbonate molar ratio (50) and dilution ratio (5.7). The resorcinol and carbonate were dissolved before the combination with BA and heated to 80 °C for 30 min to form the gel. After gel formation, it was left 24 h to form a network, the temperature was increased for 2 h to 60 °C and then maintained for an additional 2 h at 100 °C. The resulting aqueous gels were then frozen and dried for at least 48 h using a freeze dryer (Telstar-LyoQuest). The synthesis of black liquor-based aerogels involved replacing black liquor (BL) with 50% resorcinol, and due to its alkalinity, the polycondensation reaction was carried out without sodium carbonate. The gels synthesized from BL1 to BL5 were labeled AGB1–AGB5.

Phosphoric acid was used to activate the dried BLs or synthesized aerogels in a horizontal tubular furnace at 450 C for 60 min, without the presence of air. The ratio of phosphoric acid to dried precursor was 3:1. The carbon nanostructures prepared from aerogels AGB1 to AGB5 were labeled as AGB1-CNSs to AGB5-CNSs.

### Characterization of carbon precursors and nanostructure

#### Thermo-gravimetric analyses

This test was carried out using Setaram LABSYS EVO STA, France. The non-isothermal thermo-gravimetric analysis (TGA) and derivative thermogravimetry (DTG) of the BLs or butyraldehyde carbon aerogels (BAGs) as precursors for CNS production is done. Using a heating scan rate of 10 °C/min and an inert environment of nitrogen gas (30 mL/min), the analysis is carried out throughout a temperature range of 30–600 °C. According to the references^29,30^, the Kinetic parameters of thermal degradation are estimated using the following equation. The thermal activation energy (E_a_) has been evaluated by applying Coat and Redfern method for the thermogravimetric analysis.

$$log\left[\frac{-\text{ln}(1-\propto )}{{T}^{2}}\right]=\text{log}\left[\frac{ZR}{\beta {E}_{a}}\left(1-\frac{2RT}{{E}_{a}}\right)\right]-\frac{{E}_{a}}{2.303 RT} \, n=1$$

$$log\left[\frac{1-{\text{ln}(1-\propto )}^{1-n}}{(1-n){T}^{2}}\right]=\text{log}\left[\frac{ZR}{\beta {E}_{a}}\left(1-\frac{2RT}{{E}_{a}}\right)\right]-\frac{{E}_{a}}{2.303 RT} \, n\ne 1$$

where α is the fraction of material decomposed at time t, k is the specific rate constant, T is the absolute temperature, Ea is the activation energy, R is real gas constant, z is the frequency factor (s^−1^) and n is the order of reaction.

#### ATR-FTIR spectra

Using a German-made VERTEX 80v-FTIR spectrophotometer, the prepared black liquors, aerogels, or their carbon nanostructure is exposed to FTIR analysis. To assign the function groups to the prepared samples, spectral data between 400 and 4000 cm^−1^ were gathered.

#### Adsorption studies

The prepared CNSs' adsorption to iodine, methylene blue (MB), an example of a cationic dye, and Coomassie brilliant blue R-250, an example of an anionic dye, is evaluated in order to assess the adsorption behavior of the CNSs in aqueous solutions.

#### Iodine value

At room temperature (25 °C), the evaluation of the iodine value was conducted. The iodine number test is regarded as the most fundamental measure to describe the development of micro-porosity in carbon-based materials. The method defined by ASTM D 4607–94 in 2006^31^ was followed to ascertain the results of the iodine value experiment. Briefly, the experiment involved adding 0.05g of CNSs to dry flask, followed by adding 25ml of 0.1N Iodine solution and 2.5ml of 5% HCl. The flask was shaken for 30 s, filtered, and collected. The filtrate was then titrated against standard sodium thiosulphate solution using starch as an indicator. A blank sample was prepared without adding CNSs.

#### Batch equilibrium studies

Adsorption tests were run in a batch mode. The dyes methylene blue (MB) and Coomassie brilliant blue R-250 (CBB) were combined to create a stock solution. In each experiment, 25 mg of CNSs were combined with 10 mL of a known dye solution. The mixture was stirred continuously for 100 rpm at a temperature of 30 °C. Filtration was used to remove the dye solutions from the adsorbent after 24 and 48 h. A UV–Vis Single Beam spectrophotometer (UV1720, USA) was used to measure the dye content in the filtered solutions. The equilibrium adsorption of the MB and CBB dyes was calculated using the absorbance at 664 nm and 553 nm^32^. The Langmuir, Freundlich, Temkin, and Dubinin-Radushkevich (D–R) isotherms^33–36^, were used to compute the MB and BB adsorption capacities at equilibrium (qe, mg/g) as follows:

$${Q}_{e}=\frac{\left({C}_{o}-{C}_{e}\right)\text{V}}{\text{W}}$$

where C_o_ and C_e_ (mg/L) are the liquid-phase concentrations of the dye at initial and equilibrium.

V (L): volume of the dye solution.

W (g): weight of CNSs.

**Table:** 

Batch model	Equation form	References
Langmuir isotherm	$\frac{{C}_{e}}{{q}_{e}}=\frac{1}{\text{b}{q}_{m}}+\frac{{C}_{e}}{{q}_{m}}$	^33^
Freundlich isotherm	${\text{log}qe=\text{log}K}_{F}+\frac{1}{n} log {C}_{e}$	^34^
Temkin isotherm	${q}_{e}= \frac{RT}{B}\text{Ln }{\text{A}}_{T} +\frac{RT}{B}\text{Ln }{\text{C}}_{T}$	^35^
D–R isotherm	$Ln {q}_{e}=Ln {q}_{m}-\beta {\varepsilon }^{2}$	^36^
	$$\varepsilon =RT Ln (1+\frac{1}{{C}_{e}})$$	
	$${E}_{DR}=\frac{1}{\sqrt{-2\beta }}$$	

#### Adsorption kinetic studies

The kinetics of the MB and CBB dyes adsorption on CNSs are studied by employing the Lagergren first-order, pseudo-second order, and intraparticle diffusion models at 500 and 50 mg/L, respectively, with time intervals ranging from 1 to 48 h. The rate equations^37–39^, that have been most frequently employed for the adsorption of an adsorbate from an aqueous solution are expressed by the equations.

**Table:** 

Kinetic model	Linear form	Plots	References
Lagergren first order	$\text{ln}\left({\text{q}}_{\text{e}}-{\text{q}}_{\text{t}}\right)={\text{lnq}}_{\text{e}}-{\text{K}}_{1}\text{t}$	$\text{ln}\left({q}_{e}-{q}_{t}\right)$ versus t	^37^
pseudo-second order	$\frac{\text{t}}{{\text{q}}_{\text{t}}}= \left[\frac{1}{{\text{K}}_{2}{\text{q}}_{\text{e}}^{2}}\right]+\frac{1}{{q}_{e}} t$	t/q_e_ versus t	^38^
Intraparticle diffusion	${\text{q}}_{\text{t}}= {\text{K}}_{\text{id }}{\text{ t}}^\frac{1}{2}+\text{C}$	q_t_ versus t^1/2^	^39^

Where q_e_ and q_t_ are the amount of dye adsorbed per unit mass of the adsorbent (in mg/g) at equilibrium time and time t, respectively, k is the rate constant, and C is the intraparticle diffusion constant.

### Effect of pH and temperature

This study was carried out on samples which achieved optimum adsorption, of MB (AGB3-CNSs) and CBB (AGB5-CNSs) starting dye concentrations of 600 mg/L for MB and 200 mg/L for CBB at 30 °C. The impact of pH on the adsorption capacity was investigated by adjusting the pH from 4 to 11. The dye solution's initial pH was adjusted by adding a 0.1M HCl or NaOH solution. The same samples with the same concentration were also subjected to the effects of temperature variations between 20 and 40 °C.

### Surface acidity and basicity

In a closed flask, 150 mg of ABG-CNSs were combined with 15 ml of either 0.1M NaOH or 0.1M HCl solution, and the mixture was shaken for 24 h at room temperature to determine the surface acidity and basicity. After filtering the suspension, 0.1M HCl or 0.1M NaOH solution was used to titrate and determine how much NaOH or HCl was left in the solution to measure the acidity and basicity of the surface carbon nanostructure.

For further evaluating the role of pulping agents on the adsorption behavior of BL-CNSs or AGB-CNSs, selected CNSs samples prepared from CNSs of pulping agents NaOH, KOH/NH_4_OH, and Na_2_SO_3_/Na_2_CO_3_ were subjected to the following analyses.

### Textural characterization

Nitrogen adsorption–desorption isotherms that were performed at 77K using the BELSORP III analyzer series in Japan were used to characterize the texture of CNSs samples made from BL samples. In a 250 °C oven for 24 h, the samples were degassed before being examined with BELMaster version 7.3.1 software. Before the measurement got to 5.267E−5 Pa, the device was set to a vacuum level. With the help of the BET, BJH, and t-plot methods, this test was carried out to determine the surface area and pore volume.

### Scanning electron microscope (SEM)

Scanning electron microscopy (SEM) was used to analyze the morphology of the selected CNSs. Using a Quanta 3D 200i-Russia system operating at 30 kV, the samples were evaluated.

## Results and discussion

### Elemental analysis of black liquors

Table 1 displays the elemental analysis of dried black liquors. These components generally match the amounts of lignin and other low-molecular-weight substances present in black liquor. The percentages of hydrogen, oxygen, and carbon in BLs range from 4.05 to 5.11%, from 16.3 to 21.2%, and from 57.85 to 66.69%, respectively. The pulping reagents NaOH, NaOH-BH, and neutral sulfite which are related to BL1, BL2, and BL4, have the largest carbon content; more than 60%. For other pulping agents, including Kraft-BL (BL5), the highest levels of lignin and silica removal are 73.11% and 64.82%, respectively. This resulted in moderately distributed carbon at 58.9% and the highest oxygen concentration at 21.2%. Specific BL samples produced from pulping by sulfur-containing reagents coded BL4 and BL5, as a result of neutral sulfite and Kraft pulping are indicative of their sulfur content 5.50 and 4.95%, respectively. While BL samples produced from using KOH/NH_4_OH pulping reagent; BL3, are indicative of their nitrogen content 12.5%. According to the aforementioned results, BLs with high carbon content moderate amounts of lignin and silica, are useful as carbon precursors or as resorcinol substitution materials in the synthesis of aerogels. The high carbon content of BL1 is significant carbon nanostructure with yield, 55.58%.

**Table 1:** CHNS-O elemental analysis in different black liquors and the removed lignin and silica from RS in BLs.

Conditions of RS-pulping	Code	Elemental analyses							Removed lignin and silica in BLs		
		C, %	H, %	N, %	S, %	O, %	H/C	O/C	Lignin removal, %	Silica removal, %	CNSs yield, %
NaOH (18.6% Na_2_O)	BL1	66.69	4.05	–	–	19.7	0.061	0.295	34.86	57.96	55.58
NaOH (18.6% Na_2_O)-3%BH	BL2	63.75	4.08	–	–	16.3	0.064	0.256	13.32	41.6	54.39
KOH/NH_4_OH (1:5)	BL3	57.85	5.11	12.51	–	20.32	0.088	0.351	17.81	15.42	38.95
Neutral sulfite (Na_2_SO_3_/Na_2_CO_3_ [4:1])	BL4	60.83	4.89	–	5.50	16.7	0.080	0.275	10.83	14.6	17.15
Kraft reagent (NaOH-Na_2_S (10% active alkali and 25% sulfidity)	BL5	58.9	5.1	–	4.95	21.2	0.087	0.360	73.11	64.82	17.16

With regard to the role of adding BH to the NaOH pulping agent it has a negligible effect on the ratio of hydrogen to carbon element; H/C ratio, of BL, where it changes from 6.2 × 10^−2^ to 6.4 × 10^−2^. Employing BL3-BL5, this value increased. The highest value of KBL; BL5; 8.8 × 10^−2^, indicates the lignin structure in black liquor, as the largest delignification% occurred;73.1%. The O/C ratios of BL3 and BL5 are higher, ~ 0.36, than other BLs. The previous observation is focused on the effects of silica content and delignification degree.

### Characterization of carbon precursors and nanostructure

#### Thermo-gravimetric analyses

**Figure 1:**
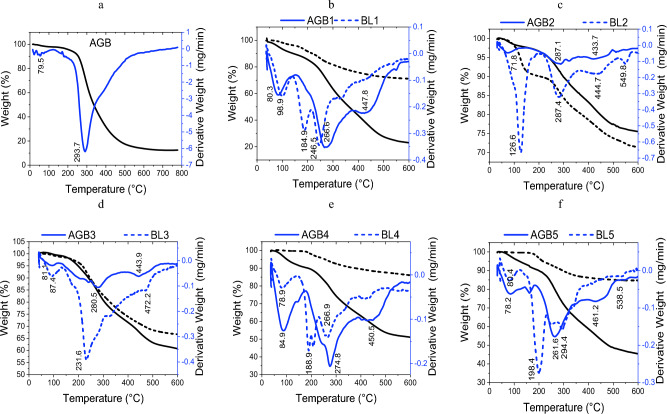
TGA of BL-resorcinol butyraldehyde carbon aerogels (AGB) versus Black liquors (BLs).

The thermal analysis of the studied BL in comparison with AGB samples as carbon precursors is shown in Fig. 1 and Tables 2, 3. According to the TGA thermograms, the evaporation of moisture content and the binding water detected with derivative thermogravimetry (DTG) at temperatures below 125 °C. Low-molecular-weight organic components found in BLs or AGB begin to breakdown between 150 and 350 °C, which indicates to the volatilization stage, while high-molecular-weight components and polymerized molecules begin to break down between 400 and 600 °C, which known carbonization stage. Based on the obtained TGA data, the BL and AGB samples can be activated and carbonized at 450 °C, as candidates for our investigation.

**Table 2:** Non-isothermal TGA measurements of AGBs free and based on BLs of RS from NaOH, NaOH-BH and KOH–NH_4_OH pulping reagents.

Sample code		Temp. range	DTG peak	Wt. remain at 450 °C, %	n order	R	Se	Ea (kJ/mol)
AGB	1^st^	50–131.2	79.5	22.58	–	–	–	–
	2^nd^	189.9–512.1	293.7		1.5	0.974	0.184	232.7
								**232.7**
AGB1	1^st^	38.7–154.2	80.3	34.2	–	–	–	–
	2^nd^	195.7–373.9	266.6		2	0.958	0.218	117.766
	3^rd^	373.6–562.2	447.8		2	0.949	0.228	187.002
								**304.768**
BL1	1st	63.7–141.8	98.8	73.78	–	–	–	–
	2nd	142.2–219.3	184.9		2	0.961	0.2	173.153
	3rd	217.8–290.4	246.5		2	0.948	0.215	227.784
								**400.937**
AGB2	1^st^	31.6–147.0	71.8	79.8	–	–	–	–
	2^nd^	206.1–379.2	287.1		2	0.959	0.213	123.407
	3^rd^	379.3–496.0	433.7		2	0.936	0.229	252.337
								**375.744**
BL2	1st	50.1–192.8	126.6	75.85	–	–	–	–
	2nd	206.5–356.5	278.4		2	0.9707	0.193	145.227
	3rd	356.4–506.6	444.6		2	0.942	0.231	196.806
	4th	521.8–576.3	549.8		2	0.951	0.206	718.612
								**1060.645**
AGB3	1^st^	39.9–118.9	81.1	67.05	–	–	–	–
	2^nd^	217.8–371.0	280.5		2	0.958	0.211	131.618
	3^rd^	375.0–534.6	443.9		2	0.953	0.222	217.286
								**348.904**
BL3	1st	40.8–131.9	87.4	70.79	–	–	–	–
	2nd	141.1–357.5	231.6		2	0.968	0.23	102.468
	3rd	431.0–507.7	472.2		2	0.939	0.219	405.773
								**508.241**

**Table 3:** Non-isothermal TGA measurements of AGBs free and based on BLs of RS from neutral sulphite and Kraft pulping reagents.

Sample code		Temp. range	DTG peak	Wt. loss, %	n Order	R	Se	Ea (kJ/mol)
AGB	1^st^	50–131.2	79.5	22.58	–	–	–	–
	2^nd^	189.9–512.1	293.7		1.5	0.974	0.184	232.7
								232.7
AGB4	1^st^	37.8–175.2	84.9	62.96	–	–	–	–
	2^nd^	175.5–383.2	274.8		2	0.966	0.217	109.379
	3^rd^	386.0–520.6	450.5		2	0.935	0.245	240.156
								**349.535**
BL4	1st	54.7–138.9	78.9	88.37	–	–	–	–
	2nd	163.4–199.6	188.9		2.5	0.968	0.232	465.951
	3rd	199.6–229.4	204.7		1.5	0.939	0.184	361.524
	4th	229.3–308.1	266.9		2	0.945	0.216	217.936
								**1045.411**
AGB5	1st	44.6–7-121.5	78.2	57.95	–	–	–	–
	2nd	183.9–369.4	261.6		2	0.968	0.208	120.363
	3rd	370.7–516.5	461.2		2	0.949	0.22	218.701
								**339.063**
BL5	1st	42.3–122.9	80.4	85.49	–	–	–	–
	2nd	152.7–229.9	198.4		2	0.972	0.213	227.420
	3rd	271.8–330.0	294.4		2	0.936	0.22	305.936
	4th	517.0–551.2	538.6		2	0.942	0.2	1127.694
								**1661.050**

Due to BL samples containing low molecular weight components like hemicellulose and lignin, they start to degrade at lower temperatures than pure aerogel or BL-resorcinol butyraldehyde carbon aerogel. The onset temperatures of aerogels are between 175 and 217 °C; while those of BLs range from 140 to 206 °C. As displayed in Fig. 1, BL samples can be degraded in two to three stages with high activation energies (Ea) that range from 400 to 1660 kJ/mol. BL1 and BL3 (NaOH and KOH/NH_4_OH) had lower Ea of 400 and 508 kJ/mol, respectively, than BL2, BL4 and BL5, which correspond to NaOH–BH, neutral sulphite, and kraft pulping agents, respectively. This indicates that adding BH to the NaOH pulping process will increase the black liquor (BL2) thermal activation energy. Additionally, the thio-lignin and sulfonyl containing lignin may be the cause of the high Ea of BL4 and BL5.

It is noticed that the aerogel in absence of BL, AGB, decomposes in one stage whereas the 2 stages decomposition are noticed for BL-substituted aerogels, AGB1–AGB5. This additional stage is probably related to the breakdown of crosslinked lignin aerogels or unreacted BL components. As seen,the degradation of BL-substituted aerogels begins at a higher temperature, 176–218 °C, than that of pure aerogel,190 °C. The major peak temperature of BL-substituted aerogels, 261–280 °C, is lower than that of pure aerogels; 293 °C. Finally, BL-aerogels have higher activation energy (Ea), ranging from 304 to 376 kJ/mol, as compared to pure aerogels, 232 kJ/mol.

#### ATR-FTIR spectra

**Figure 2:**
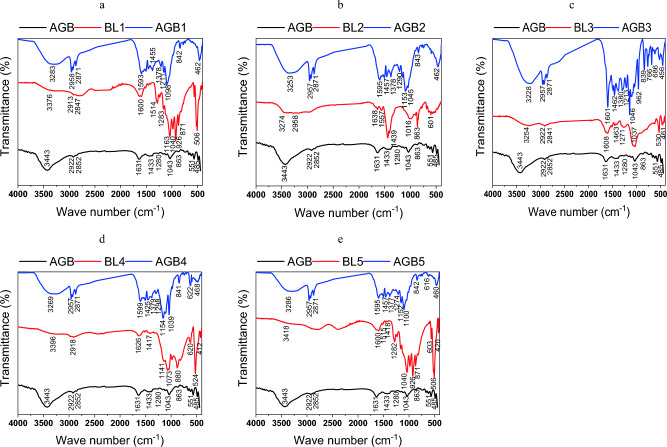
FTIR of AGBs free and based on BLs versus Black liquors (BLs).

ATR-FTIR analysis of the different pulping BLs in contrast to the unsubstituted and substituted aerogels is shown in Fig. 2a–e. As can be seen from the spectra, the majority of the lignin and silica bands are present in all BL samples derived from the various pulping processes. Broad bands assigned to the OH stretching vibrations of phenolic lignin, uncondensed –CH_2_OH groups, carboxylic acid, or adsorbed water are observed at 3250–3460 cm^−1^. Low-intensity bands are attributed to CH asymmetric stretching at 2940 cm^−1^. A band at 1650 cm^−1^ and 1550 cm^−1^ is used to assign the aromatic skeletal vibration (C=C) of lignin (guaiacyl or syringyl) in BLs. In the range of 800–873 cm^−1^, the benzene ring of the lignin group exhibits stretching and bending as well^40^. The C=O stretching (non-conjugated ketones or carbonyl groups), CH deformation in CH_3_ or CH_2_, or phenyl propane skeleton vibrational absorption bands, as well as the ether group C–O–C linkage, are assigned by the bands at 1650, 1440, and 1210 cm^−1^, respectively. Bands in areas 850–1050 cm^−1^ for Si–O–Si asymmetric stretching and 400–800 cm^−1^ for Si–O–Si bending highlight the presence of silica^10,41^.

In contrast to the aerogels produced from resorcinol and butyraldehyde, AGB ,the FTIR spectra of aerogels from BL-resorcinol/butyraldehyde, AGB1–5, the spectra show that the bands associated with OH stretching vibrations of BL-based aerogels are red shifted from 3443 cm^−1^ to wave number range 3228–3286 cm^−1^ with degree of change ranging Δν215–157 cm^−1^.The substituted aerogels with BL from NaOH–BH and KOH/NH_4_OH pulping, AGB2 and AGB3, exhibit the highest degree of shifting, Δν190 and 215, these values correlating to their highest activation energy which reach 376 and 349 kJ/mol, respectively. The peak intensity at 1000–1100 is increased in comparison to pure aerogel when the resorcinol was replaced with BLs in the aerogel. Additionally, due to the presence of BLs' silica, strong peaks are seen in all aerogels' fingerprint regions at 600–800 cm^−1^, especially in AGB3. For CH asymmetries of 2871, 2930, and 2957 cm^−1^ all substituted aerogels exhibit three distinct bands.

### Adsorption behavior

#### Iodine value

**Figure 3:**
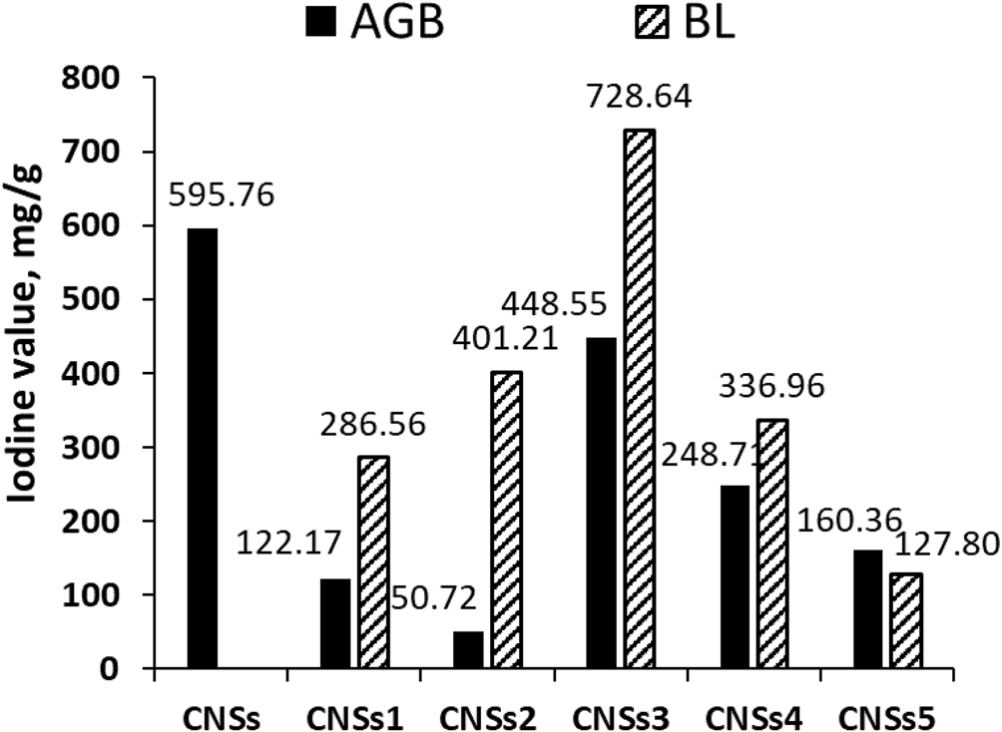
Iodine value of CNSs from AGBs free and based on BLs versus Black liquors (BLs).

The iodine adsorption values on the surface of carbon nanostructures produced in absence and presence of BL-aerogels, AGB- and AGB1-5-CNSs, as opposed to black liquors, BL-CNSs, are shown in Fig. 3. Contrarily, the iodine value of CNSs from pure aerogels, substituted aerogels, and black liquor are 595.7, 50.7–448.6, and 122.2–728.6 mg/g, respectively. The BL3-CNSs have the greatest capacity to remove iodine from aqueous solution. The substituted aerogel with the same black liquor, AGB3, follows the same pattern and has the greatest iodine value, 448.6 mg/g, when compared to other BLs substituted aerogels.

Our results surpass those reported in many literatures. Saka^42^, investigated the production of activated carbon from acorn shell by chemical activation with zinc chloride and reported the iodine number in the range of 37–1209 mg/g; Mopoung et al.^43^, reported that the iodine adsorption of tamarind seed-based activated carbon with KOH activation ranged from 150 to 300 mg/g; Limet et al.^44^, found the iodine number of oil palm trunk was 500–880 mg/g and Liu et al.^45^, found that the iodine number of H_3_PO_4_-activated hydrochar at 600–1000 °C temperature was 200–830 mg/g. According to the iodine values presented above, black liquors or aerogels made from BL-resorcinol/butyraldehyde are effective precursors for carbon nanostructures that are mostly mesoporous in nature.

#### Dyes adsorption behavior

**Figure 4:**
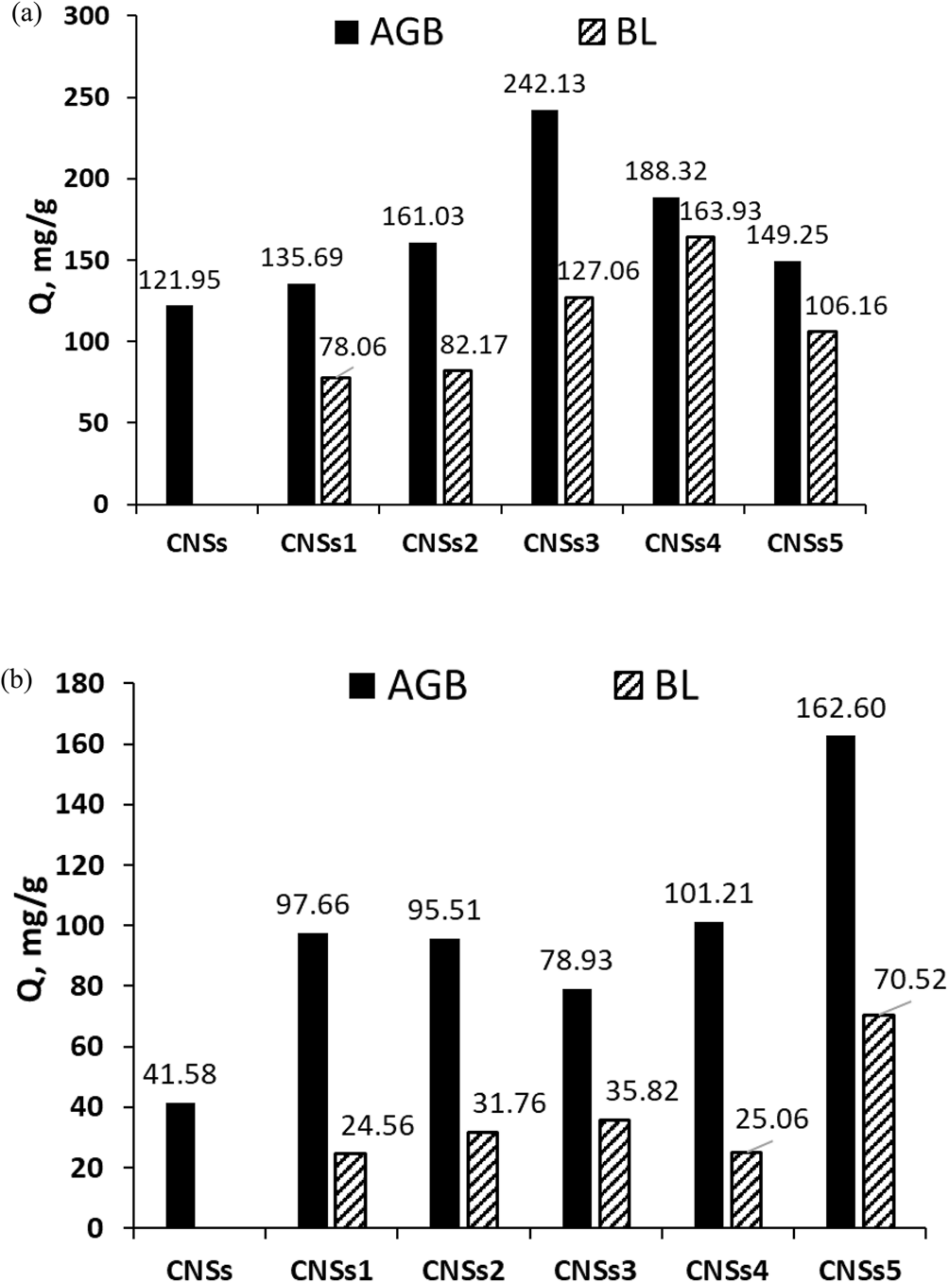
Adsorption capacity of (**a**) MB dye and (**b**) CBB dye on CNSs from AGBs free and based on BLs (AGB) versus Black liquors (BLs).

Figure 4a,b shows the impact of initial concentrations of methylene blue (MB) and Coomassie brilliant blue R-250 (CBB) dyes on the adsorption capacity of the CNSs to evaluate and compare the contribution of BLs to performance of the produced aerogel or BL itself as effective precursor for development of CNSs. According to the findings, the BL-substituted aerogels' entire carbon nanostructure with a better effect on eliminating MB and CBB than BL-CNSs and pure aerogel-CNSs, where its capacity is 121.9 for MB and 41.6 for CBB mg/g. To produce CNSs with a high adsorption capacity, 242.1 mg/g, the aerogels from BLs of alkaline pulping (KOH/NH_4_OH) coded AGB3-CNSs recommend as precursors, followed by those made from neutral pulping AGB4-CNSs; 188.32 mg/g. Comparing black liquor CNSs to pure aerogel the BL3 and BL4 have the highest MB adsorption capacities with values 127.06 and 163.93 mg/g, respectively.

Figure 4b shows the CNSs made from BL-aerogel of Kraft pulping, AGB5, precursor has an effective role in the removal of Coomassie Brilliant Blue, CBB, as example to anionic dye, the removal reaches 162.6 mg/g. Using this BL5 as precursor of CNSs also provides the highest adsorption capacity reaches to 70.5 mg/g.

The parameters registered in Tables 4 and 5 are related to the MB and CBB adsorption capacity at equilibrium by applying the isotherms of Langmuir, Freundlich, Temkin, and dubinin–radushkevich (D–R). Based on the correlation coefficient (R^2^) for the elimination of MB dyes, it can be shown that the Langmuir model offers a better match (R^2^) than other models that have been looked at for all analyzed CNSs. The (R^2^) value lay between 0.99 and 1.00. Furthermore, the measured R_L_ values between 0 and 1 demonstrated that the produced CNSs were advantageous for MB dye adsorption under the circumstances employed in this study. This indicates that the MB adsorption is valid for the monolayer adsorption on homogenous surface. All the samples fit the Freundlich isotherm, which is valid for heterogeneous surfaces, well for CBB adsorption on the examined CNSs. The adsorption of CBB on carbon-based materials fits well to the Freundlich isotherm, in agreement with^32,46^. The high value of n, 0.74–36.2, points to a robust interaction between the surface of the carbon nanostructure and the CBB dye. With R^2^ at 0.98 and R_L_ value 0.027, the Langmuir isotherm model fits BL7-CNSs the best. A comparison of our investigated work maximum capacity results of MB and CBB with previously reported findings is given in Tables 6 and 7 respectively^47–54^.

**Table 4:** Langmuir, Freundlich, Temkin and dubinin–radushkevich (D–R) isotherm parameters for adsorption of MB dye onto AGBs free and based on BLs-CNSs versus BL-CNSs.

		Adsorbents										
Model	Isotherm parameters	AGB-CNSs	AGB1-CNSs	BL1-CNSs	AGB2-CNSs	BL2-CNSs	AGB3-CNSs	BL3-CNSs	AGB4-CNSs	BL4-CNSs	AGB5-CNSs	BL5-CNSs
Langmuir isotherm	Q_m_, mg g^−1^	121.9	135.6	78.06	161.0	82.16	242.1	127.0	188.3	163.9	149.2	106.1
	b, L mg^−1^	0.759	13.90	1.306	0.33568	0.086	0.94943	0.966	1.28883	0.216	0.66337	1.954
	R_L_,mg L^−1^	0.0022	0.00012	0.0013	0.0049	0.0191	0.0017	0.0017	0.0013	0.0076	0.0025	0.0009
	R^2^	0.999	0.994	0.993	0.9969	0.967	0.9979	1.000	0.9998	1.000	0.9988	1.000
Freundlich isotherm	N	5.157	4.420	6.452	5.704	4.505	2.823	4.808	5.293	3.521	6.882	5.208
	K_F_,mg g^−1^	57.22	50.68	39.90	69.63	30.43	100.2	64.26	91.18	48.18	75.61	61.40
	R^2^	0.878	0.802	0.570	0.798	0.576	0.845	0.657	0.823	0.831	0.702	0.736
Temkin isotherm	B, J.mol^−1^	4.223	0.130	0.283	0.153	0.414	0.061	0.174	0.127	0.107	0.188	0.193
	K_T_, L.mg^−1^	68.24	15.27	95.09	110.8	1642.8	17.96	126.4	203.7	7.579	548.4	165.8
	R^2^	0.954	0.754	0.538	0.908	0.870	0.928	0.800	0.936	0.935	0.827	0.774
D–R isotherm	Β, mol^2^ KJ^−2^	1E−07	2E−07	2.0E−06	1E−07	7.0E−06	7E−08	2.0E−07	4E−08	8.0E−07	8E−08	7.0E−08
	Q_m_, mg g^−1^	107.5	122.7	81.6	139.8	76.87	184.0	119.4	161.1	145.4	144.2	96.62
	E_D–R_, KJ mol^−1^	2.236	1.581	0.500	2.236	0.267	2.672	1.581	3.535	0.791	2.500	2.673
	R^2^	0.942	0.762	0.947	0.949	0.787	0.904	0.803	0.947	0.956	0.947	0.943

**Table 5:** Langmuir, Freundlich, Temkin and dubinin-radushkevich (D–R) isotherm parameters for adsorption of CBB dye onto AGBs free and based on BLs-CNSs versus BL-CNSs.

Model	Isotherm parameters	Adsorbents										
		AGB-CNSs	AGB1-CNSs	BL1-CNSs	AGB2-CNSs	BL2-CNSs	AGB3-CNSs	BL3-CNSs	AGB4-CNSs	BL4-CNSs	AGB5-CNSs	BL5-CNSs
Langmuir isotherm	Q_m_, mg g^−1^	41.58	97.65	24.55	95.51	31.75	78.92	35.81	101.2	2.506	162.602	70.52
	b, L mg^−1^	0.015	0.010	0.009	0.018	0.009	0.046	0.024	0.014	0.146	0.011	0.060
	R_L_,mg L^−1^	0.101	0.148	0.154	0.087	0.150	0.035	0.065	0.105	0.011	0.882	0.027
	R^2^	0.983	0.994	0.515	0.979	0.977	0.930	0.845	0.839	0.002	0.137	0.976
Freundlich isotherm	N	36.23	11.82	1.636	2.976	0.583	1.446	1.547	3.259	0.735	0.856	1.275
	K_F_,mg g^−1^	1.066	1.215	1.511	2.168	150.557	4.917	1.280	2.027	7.698	2.035	3.112
	R^2^	0.985	0.996	0.914	0.996	0.956	0.991	0.917	0.999	0.917	1.000	0.791
Temkin isotherm	B, J.mol^−1^	0.350	0.205	0.646	0.187	0.351	0.212	0.381	0.195	0.156	0.159	0.157
	K_T_, L.mg^−1^	3.910	3.660	5.028	2.294	12.055	1.145	3.028	2.427	7.921	2.367	1.750
	R^2^	0.945	0.905	0.769	0.903	0.951	0.863	0.791	0.848	0.986	0.859	0.969
D–R isotherm	Β, mol^2^ KJ^−2^	6.E−06	4.E−06	1.0E−05	2.E−07	7.0E−05	3.E−07	4.0E−06	2.E−06	4.0E−05	2.0E−06	3.0E−06
	Q_m_, mg g^−1^	15.19	20.96	8.65	23.78	10.57	24.82	11.60	22.30	31.50	25.860	42.73
	E_D-R_, KJ mol^−1^	0.289	0.354	0.224	1.581	0.085	1.291	0.354	0.500	0.112	0.500	0.408
	R^2^	0.771	0.720	0.765	0.692	0.886	0.635	0.133	0.655	0.967	0.706	0.953

**Table 6:** Adsorption capacities of present investigated CNSs to MB versus literature.

Case study	Q for MB, mg/g	Carbonaceous materials	Q for MB, mg/g	References
AGB	121.95	CNTs from carbon xerogel	80.1–88.1	^21^
AGB1-CNSs	135.69	Carbon xerogel/titania composites	255	^47^
AGB2-CNSs	161.03	MnO_2_ modified lignin biochar	248.96	^45^
AGB3-CNSs	242.13	Coconut shell activated carbon	277.9	^48^
AGB4-CNSs	188.32	Bamboo dust activated carbon	143.2	^48^
AGB5-CNSs	149.25	Amino-functionalized cellulose nanofiber/lignosulfonate aerogel	170.94	^49^
BL1-CNSs	78.06	KOH-activated resorcinol–formaldehyde carbon gels	135	^50^
BL2-CNSs	82.17			
BL3-CNSs	127.06			
BL4-CNSs	163.93			
BL5-CNSs	106.16

**Table 7:** Adsorption capacities of present investigated CNSs to CBB versus literature adsorbents.

Case study	Q for CBB, mg/g	Carbonaceous materials	Q for CBB, mg/g	Reference
AGB	41.58	Poly(phenylenediamine) grafted electrospun carbon nanofibers (PPDA-*g*ECNFs)	6–100 mg/g	^13^
AGB1-CNSs	97.66	Active carbon from Nigella sativa waste	14.49 mg/g	^32^
AGB2-CNSs	95.51	Copper Oxide/Carbon nanocomposites from Vitex negundo Linn leaf	9.09 mg/g	^51^
AGB3-CNSs	78.93	Iron-pillared bentonite	9.125	^52^
AGB4-CNSs	101.21	Sodium bentonite	6.848	^52^
AGB5-CNSs	162.60	Iron oxide–graphene oxide composite	14.31 mg/g	^53^
BL1-CNSs	24.56	goat dropping activated carbon	270.3 mg/g	^54^
BL2-CNSs	31.76			
BL3-CNSs	35.82			
BL4-CNSs	25.06			
BL5-CNSs	70.52			

#### Batch kinetic studies

The kinetic parameters for the removal of dyes using the models Lagergren first-order, pseudo second order, and intraparticle diffusion are shown in Tables 8 and 9. The pseudo-second order model is more relevant based on the maximum correlation coefficient (R^2^) and minimum standard error estimate (SEE) values, R^2^ 0.99–1.00, SEE 0.001–2.440. In comparison to the first order and intraparticle diffusion models, the pseudo-second order model has lower Standard Error of Estimate (SEE) values. The pseudo-second-order kinetics model was the most fit model to the adsorption behavior of both dyes on both CNSs (aerogels and BLs), and the results suggest that chemisorption may be the adsorption mechanism and that the adsorption kinetics is dominated by adsorption onto the active site^55^.

**Table 8:** Lagergren first order, pseudo second order and intraparticle diffusion kinetics parameters for MB dye adsorption onto AGBs free and based on BLs-CNSs versus BL-CNSs.

Model	Kinetic parameters	Adsorbents										
		AGB-CNSs	AGB1-CNSs	BL1-CNSs	AGB2-CNSs	BL2-CNSs	AGB3-CNSs	BL3-CNSs	AGB4-CNSs	BL4-CNSs	AGB5-CNSs	BL5-CNSs
Lagergren first order model	K_1_, h^−1^	0.028	0.008	0.040	0.008	0.035	0.007	0.041	0.016	0.053	0.001	0.082
	Q_eq_, mg g^−1^	361.9	386.4	52.055	378.0	52.107	315.8	46.344	368.0	53.936	378.0	42.470
	R^2^	0.979	0.965	0.942	0.906	0.940	0.933	0.853	0.984	0.897	0.725	0.954
	SEE	0.035	0.015	0.057	0.013	0.038	0.013	0.056	0.010	0.061	0.013	0.074
Pseudo-second order	K_2_, mg g^−1^	0.004	0.015	0.039	0.013	0.003	0.041	0.021	0.016	0.004	0.019	0.006
	Q_eq_, mg g^−1^	236.4	145.1	9.787	152.0	21.993	199.2	17.775	170.9	26.069	138.9	40.634
	R^2^	0.995	1.000	0.995	1.000	0.959	1.000	0.997	1.000	1.000	1.000	0.993
	SEE	0.001	0.001	2.440	0.001	2.288	0.001	0.032	0.000	2.301	0.001	0.020
Intraparticle diffusion	K_id_, mg g^−1^ h^−1/2^	18.819	4.893	6.089	4.430	3.597	7.850	2.534	19.220	4.826	3.468	6.082
	C, mg g^−1^	136.2	114.8	6.399	123.8	3.082	177.9	4.471	117.8	4.442	116.8	6.216
	R^2^	0.940	0.907	0.946	0.908	0.957	0.976	0.823	0.979	0.927	0.805	0.968
	SEE	8.262	3.727	2.215	3.353	1.168	3.353	1.824	2.594	2.105	4.067	1.725

**Table 9:** Lagergren first order, pseudo second order and intraparticle diffusion kinetics parameters for CBB dye adsorption onto AGBs free and based on BLs-CNSs versus BL-CNSs.

Model	Kinetic parameters	Adsorbents										
		AGB-CNSs	AGB1-CNSs	BL1-CNSs	AGB2-CNSs	BL2-CNSs	AGB3-CNSs	BL3-CNSs	AGB4-CNSs	BL4-CNSs	AGB5-CNSs	BL5-CNSs
Lagergren first order model		0.017	0.029	0.040	0.012	0.035	0.042	0.041	0.029	0.053	0.029	0.082
	K_1_, h^−1^	42.875	46.805	52.055	45.925	52.107	44.657	46.344	43.207	53.936	42.564	42.470
	Q_eq_, mg g^−1^	0.930	0.929	0.942	0.952	0.940	0.952	0.853	0.894	0.897	0.867	0.954
	R^2^	0.024	0.037	0.057	0.026	0.038	0.055	0.056	0.041	0.061	0.043	0.074
Pseudo-second order	SEE	0.161	0.023	0.039	0.006	0.003	0.032	0.021	0.053	0.004	0.080	0.006
	K_2_, mg g^−1^	11.377	14.857	9.787	20.942	21.993	18.096	17.775	15.451	26.069	14.747	40.634
	Q_eq_, mg g^−1^	1.000	0.995	0.995	0.972	0.959	1.000	0.997	0.999	1.000	0.999	0.993
	R^2^	0.012	0.046	2.440	0.124	2.288	0.015	0.032	0.010	2.301	0.011	0.020
Intraparticle diffusion	SEE	2.295	2.119	6.089	3.021	3.597	5.508	2.534	3.929	4.826	3.858	6.082
	K_id_, mg g^−1^ h^−1/2^	5.401	3.467	6.399	0.630	3.082	1.357	4.471	3.812	4.442	4.459	6.216
	C, mg g^−1^	0.954	0.884	0.946	0.955	0.957	0.965	0.823	0.940	0.927	0.910	0.968
	R^2^	0.829	1.190	2.215	1.009	1.168	1.720	1.824	1.289	2.105	1.434	1.725

### Effect of pH and temperature

**Figure 5:**
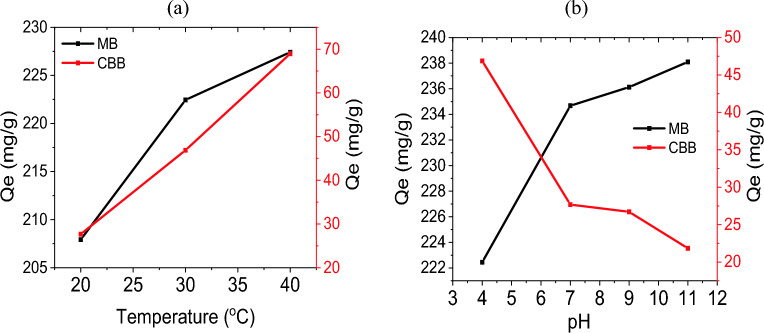
Adsorption capacity of MB and CBB on AGB-CNSs (**a**) effect of temperature (**b**) effect of pH.

The adsorption capacity of MB by ACB3-CNSs was higher in alkaline pH values, as Fig. 5a illustrates. This is because the negatively charged adsorbent surface facilitates the positively charged MB molecules. When the pH rose, the adsorption capacity grew progressively until reaching a maximum at 11. Adoption of anionic dye CBB showed a reversal trend because the negatively charged adsorbent surface increased the anionic dye's repulsion forces and decreased the adsorption capacity at high pH levels.

As the temperature rises from 20 to 40 °C, the adsorption of both dyes (MB and CBB) increases significantly (Fig. 5b). The increase in temperature causes the large dye ions to become more mobile and less swollen, which allows the large dye molecule to penetrate and increase adsorption capability of the carbon nanostructure. Additionally, the outcomes showed that MB and CBB adsorption is an endothermic process.

### Surface acidity and basicity

Various heteroatoms from the raw material or activating agent primarily influence the surface chemistry of activated carbons. The acidic nature of activated carbon is attributed to heteroatoms, such as carboxyl, carbonyl, lactone, phenol, and other bound surface functional groups. Table 10 presented the surface functional group in ABG-CNSs by the Boehm technique. The total acidity of AGB-CNSs ranges from 6.32 to 8.39 (mmol/g) based on the Boehm titration data (Table 10). It was discovered that the surface of the carbon nanostructure had more total acidity than total base. When H_3_PO_4_ is used to produce activated carbon, the carbon's surface may develop acidic surface functional groups associated with the oxygen-containing groups. On comparing the obtained data with other studies, the total acidity of our case is exceeded the other activated carbons (1.27 and 5.60 mmol/g)^56,57^ as recorded in Table 10 and as high as the oxidized activated by nitric acid (1.33–14.25 mmol/g)^58–60^.

**Table 10:** Total acidity and the base amount of AGBs based on BLs (AGB) determined by the Boehm method.

	Total acidity mmol/g	Total base mmol/g	References
AGB1-CNSs	8.39	0.20	Present work
AGB2-CNSs	7.89	0.79	
AGB3-CNSs	6.81	0.69	
AGB4-CNSs	6.32	1.48	
AGB5-CNSs	7.60	0.69	
Active carbon	1.27	1.61	^56^
Active carbon	5.60	6.22	^57^
HNO_3_ oxidized activated (69% HNO3)	14.25	–	^58^
HNO_3_ oxidized activated (0.7 M HNO3)	1.33	0.17	^59^
HNO_3_ oxidized activated (0.7 M HNO3)	3.02	1.07	^60^

### ATR-FTIR spectra of CNSs

**Figure 6:**
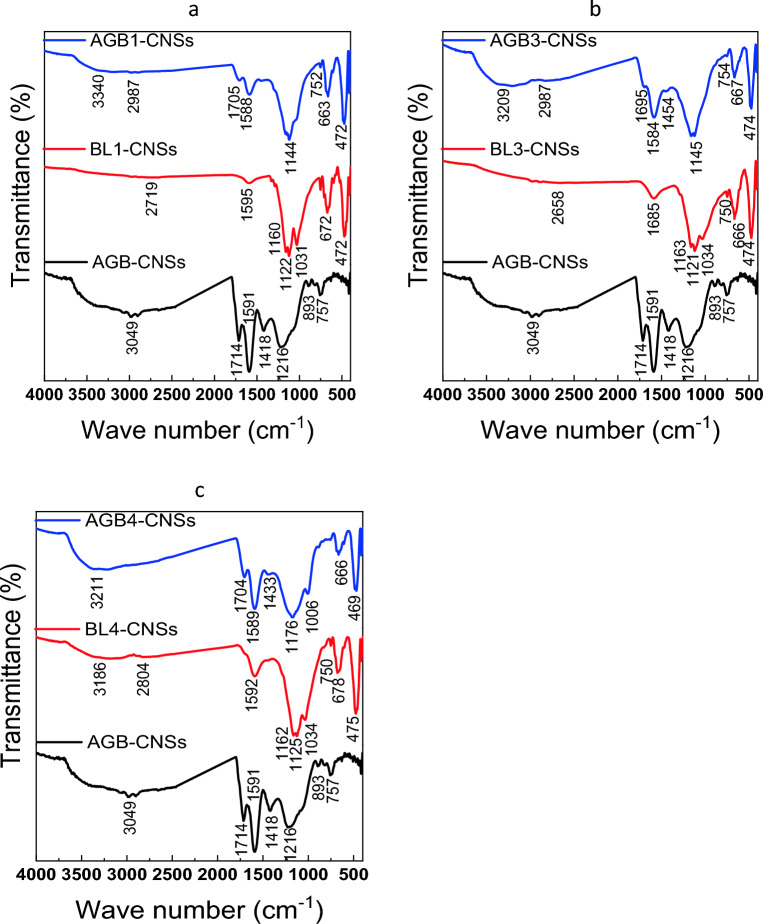
FTIR-ATR of selected AGB-CNSs versus BL-CNSs.

Based on the adsorption data and thermal analysis, some CNS samples prepared from BLs and their corresponding aerogels of various pulping agents, namely NaOH, KOH/NH_4_OH, and Na_2_SO_3_/Na_2_CO_3_ are chosen for further examination of the impact of BL pulping on functional groups included the resultant CNSs. The adsorption and thermal stabilities of these materials vary widely. The studied FT-IR spectra of CNSs are shown in Fig. 6a–c. Broad bands between 3200 and 3400 cm^−1^ are attributed to the hydrogen-bonded –OH stretching vibration of water molecules adsorbed on the carbon surface^61^. According to References^61,62^, the peak in the range 1580 and 1705 cm^−1^ is designed as a C=C stretching vibration or C=O group. O–C–O asymmetric stretching vibration is attributed to the sharp band between 1000 and 1100 cm^−1^^63^. Because the pulping process removes silica from RS and results in greater silica content in BLs, the sharpness of the ether band is related to lignin, hemicellulose, or Si–O–Si stretching^64^. The benzene ring stretching and bending at 600–700 cm^−1^ and the sharp bands at 400–500 cm^−1^ are attributed to Si–O–Si bending.

The findings demonstrate that the frequency of the OH broad band of AGB-CNSs increases from 3049 cm^−1^ to 3209–3340 cm^−1^in the case of using aerogels AGB1 AGB3 and AGB4. Additionally, the bands of carbonyl group's intensity at 1700 and 1600 cm^−1^as well as the emergence of a band at 470 cm^−1^ decreased because of the BLs substitution's included silica (Table 1). The spectra of BL-CNSs, also reveal a broadening of the –OH band, with a decrease in the frequency of the OH band. A rise in the frequency of the band associated to the Si–O–Si bending, indicates a larger silica content. Thus, negatively charged carboxyl, hydroxyl, and silicate groups are present on the carbon nanostructure surface of BLs and BL-based aerogels, which motivates the cationic dye adsorption on these active sites.

### Textural characterization of CNSs

**Figure 7:**
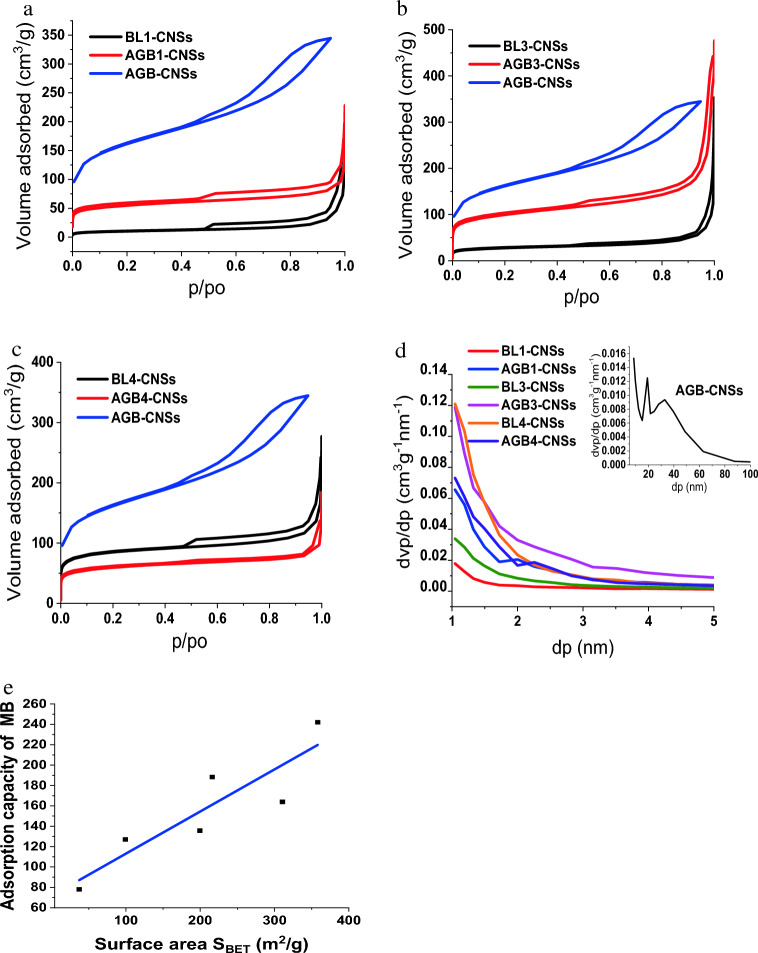
Nitrogen isotherms at 77 K of selected AGB-CNSs versus BL-CNSs from pulping BLs using different reagents (**a**) NaOH, (**b**) KOH/NH_4_OH, (**c**) Na_2_SO_3_/Na_2_CO_3,_ (**d**) Pore volume distribution and (**e**) S_BET_ and adsorption capacity relationship.

The nitrogen adsorption isotherms of the prepared AGB-CNSs in comparison with BL-CNSs is illustrated in Fig. 7a–c, while their pore volume distribution and S_BET_ and adsorption capacity relationship are shown in Fig. 7d,e. The nitrogen adsorption–desorption at 77K. N_2_ isotherms (Fig. 7a–c) display hybrid Type I–IV isotherms with H4 hysteresis, in accordance with the IUPAC classification^65,66^. These isotherms were distinguished by a significant rise in N_2_ absorption at extremely low pressures, up to relative pressures of 0.1, which suggested the presence of microporosity. Indicating the presence of mesopores since the hysteresis loop occurs between P/P0 = 0.50–0.99, this is followed by a constantly increasing N_2_ adsorbed quantity (plateau form) in the region of medium to high relative pressures. According to the findings, CNSs are made up of a variety of microspores and mesopores in varying amounts.

The results of this study are presented in Table 11, including the carbon yield C, surface area S_BET_, product C x S_BET_, S_micro_ and V_T_, micropore V_DR_, mesopore V_meso_ volumes, and finally the surface area fraction S_micro_/S_BET_. As shown, the carbon yield of BL-CNSs ranges from 17.2 to 55.6%, while those of AGB-CNSs is between 33.4 and 47.1%. The S_BET_ ranges from 37.0 to 566.5 m^2^/g. The resorcinol/butyraldehyde aerogel-CNSs, AGB, has greater BET surface areas, 566.5 m^2^/g, than alternative BL-resorcinol/butyraldehyde aerogels-CNSs, 199.4–358.1 m^2^/g. The S_BET_ of AGB1,3,5-CNSs are higher than surface of the CNSs from their corresponding BLs, which in the range 37.0–310.6 mg/g. It should be noted that the AGB3-CNSs with S_BET_358.1 m^2^/g,is distinguished by high surface area together with total pore volume, 0.548 cm^3^/g, and accepted micro surface area fraction, 58.5% (Fig. 7d, Table 11). Its behaviour contributed to its high iodine value and MB adsorption capacity. The performance of MB adsorption on selected CNSs BL1, 3, 4 and their AGBs is virtually directly related to specific surface area (S_BET)_, as shown in Fig. 7e. The BL of KOH/NH_4_OH pulping liquors induced in the aerogels are the best for making highly effective carbon nanostructure, according to the textural characterization.

**Table 11:** The pore structure parameters of selected AGB-CNSs versus BL-CNSs.

Code	Carbon yield (C) %	S_BET,_ m^2^/g	C x S_BET_ m^2^/g	V_T (0.95)_, cm^3^/g	pore diameter, nm	d_p peak,_ nm	S_micro (t-plot)_, m^2^/g	V_micro (t-plot)_, cm^3^/g	V_meso_, cm^3^/g	S_micro_/S_BET_, %
AGB-CNSs	49.90	566.5	282.9	0.533	18.82	1.045	286.3	0.120	0.410	50.49
BL1-CNSs	55.59	37.02	20.58	0.109	11.81	1.050	36.52	0.049	0.060	98.65
AGB1-CNSs	33.22	199.4	66.24	0.165	3.307	1.045	40.21	0.066	0.099	20.17
BL3-CNSs	38.96	99.23	38.66	0.165	6.646	1.045	63.66	0.008	0.157	64.15
AGB3-CNSs	41.11	358.1	147.2	0.548	6.126	1.045	209.5	0.028	0.520	58.50
BL4-CNSs	17.15	310.6	53.27	0.243	3.127	1.015	59.8	0.093	0.150	19.25
AGB4-CNSs	47.05	216.1	101.7	0.150	2.773	1.045	39.54	0.065	0.085	18.30

### Scanning electron microscope (SEM)

**Figure 8:**
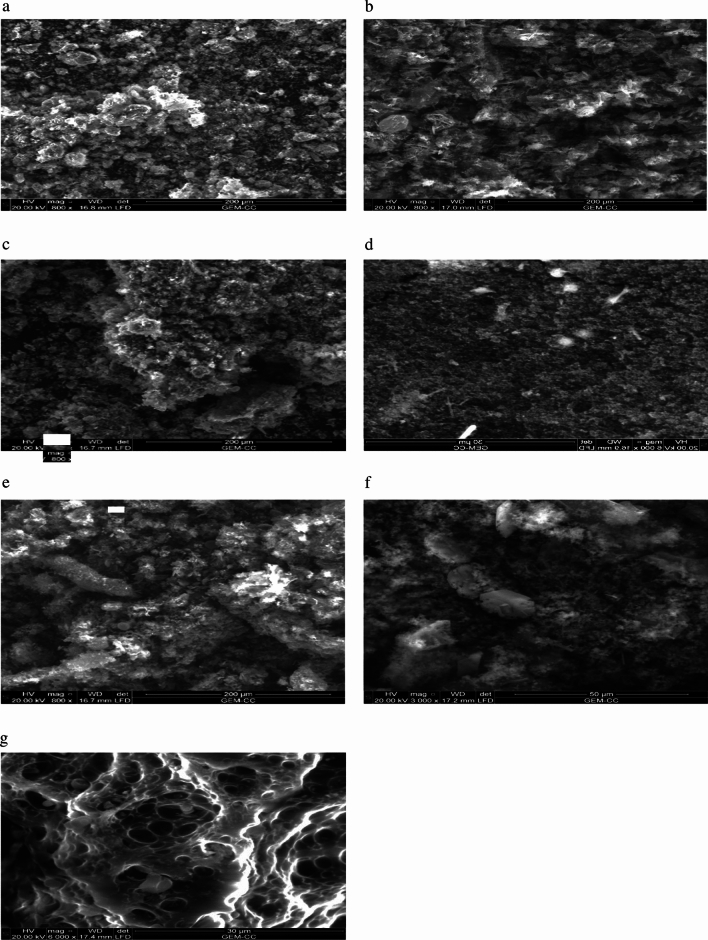
SEM micrographs of CNSs from of selected AGB-CNSs versus BL-CNSs (**a**) BL1-CNSs, (**b**) AGB1-CNSs, (**c**) BL3-CNSs, (**d**) AGB3-CNSs, (**e**) BL4-CNSs, (**f**) AGB4-CNSs and (**g**) AGB-CNSs.

Scanning electron micrographs (Fig. 8a–g) are used to demonstrate the morphology of the studied CNSs that were chosen, each having a distinct surface area. CNSs made from resorcinol/butyraldehyde aerogel precursors are depicted in Fig. 8g, whereas those made from black liquor and black liquor-resorcinol/butyraldehyde aerogels are presented in Fig. 8a–f. After H_3_PO_4_ activation of AGB, a porous structure with variable pore diameters, inhomogeneous surface, and roughness are observed. The micrographs (Fig. 8b, d, and f) demonstrate the existence of silico-phosphate crystals made of silica that were dissolved during the pulping of rice straw in black liquor when comparing the CNSs from BL-based aerogels to free aerogel. The micrographs (8a, c, and e) relate to CNSs from black liquors precursors that have increased silico-phosphate crystal content. This finding is supported by the decrease in ash content of RS pulps (Table 1) and is connected to the silica content of black liquors relative to BL-based aerogels.

## Conclusion

The study demonstrates the successful synthesis of novel aerogels by replacing resorcinol in resorcinol/butyraldehyde with black liquors of RS. The carbon nanostructure adsorbents (CNSs) were successfully synthesized, with the best-prepared AGB-CNSs produced from RS-BL using KOH/NH_4_OH reagent for cationic adsorption. The kraft BL-aerogel-CNSs was the best adsorbent against anionic dye. The Freundlich and Langmuir models were well-suited for describing the CBB and MB adsorption equilibrium, while the pseudo-second-order kinetic model successfully described both the MB and CBB adsorption processes. The study revealed that the affinity of CNSs for dye removal was significantly enhanced by the presence of functional groups (hydroxyl, silicate, carboxyl, and π–π) and active sites. The data proved the alkaline pH is more effective on adsorption capacity of MB; while the reverse trend is observed toward the CBB dye. Both dyes have the same adsorption trend (improved) against increasing the temperature. The total acidity of our investigated CNSs exceeded the literature activated carbons.

## Data Availability

All data generated or analyzed during this study are included in this article.
